# Wireless power transfer-based eddy current non-destructive testing using a flexible printed coil array

**DOI:** 10.1098/rsta.2019.0579

**Published:** 2020-09-14

**Authors:** Lawal Umar Daura, GuiYun Tian, Qiuji Yi, Ali Sophian

**Affiliations:** 1 School of Engineering, Newcastle University, Newcastle upon Tyne NE1 7RU, UK; 2 Electrical Engineering Department, Faculty of Engineering, Bayero University, Kano, Nigeria; 3 School of Automation Engineering, University of Electronic Science and Technology, Chengdu, People's Republic of China; 4 Department of Mechatronics Engineering, Faculty of Engineering, International Islamic University Malaysia, Kuala Lumpur, Malaysia

**Keywords:** eddy current testing, flexible coil array, feature extraction, selection and fusion, resonant frequency, wireless power transfer

## Abstract

Eddy current testing (ECT) has been employed as a traditional non-destructive testing and evaluation (NDT&E) tool for many years. It has developed from single frequency to multiple frequencies, and eventually to pulsed and swept-frequency excitation. Recent progression of wireless power transfer (WPT) and flexible printed devices open opportunities to address challenges of defect detection and reconstruction under complex geometric situations. In this paper, a transmitter–receiver (Tx–Rx) flexible printed coil (FPC) array that uses the WPT approach featuring dual resonance responses for the first time has been proposed. The dual resonance responses can provide multiple parameters of samples, such as defect characteristics, lift-offs and material properties, while the flexible coil array allows area mapping of complex structures. To validate the proposed approach, experimental investigations of a single excitation coil with multiple receiving coils using the WPT principle were conducted on a curved pipe surface with a natural dent defect. The FPC array has one single excitation coil and 16 receiving (Rx) coils, which are used to measure the dent by using 21 C-scan points on the dedicated dent sample. The experimental data were then used for training and evaluation of dual resonance responses in terms of multiple feature extraction, selection and fusion for quantitative NDE. Four features, which include resonant magnitudes and principal components of the two resonant areas, were investigated for mapping and reconstructing the defective dent through correlation analysis for feature selection and feature fusion by deep learning. It shows that deep learning-based multiple feature fusion has outstanding performance for 3D defect reconstruction of WPT-based FPC-ECT.

This article is part of the theme issue ‘Advanced electromagnetic non-destructive evaluation and smart monitoring’.

## 1. Introduction

The eddy current testing (ECT) has been developed for various non-destructive testing and evaluation (NDT&E) applications, such as defect detection, thickness, coating and conductivity measurements for material identification; heat damage detection; case depth determination and heat treatment monitoring. In its development, the ECT has metamorphosed through different stages, namely single-frequency [1], multiple-frequency [2], swept-frequency ECT [3,4] and pulsed or transient ECT [1,5]. In terms of the probe design, recently the ECT has adopted the use of flexible arrays [6–8].

A single-frequency ECT [1] uses a single-frequency excitation which inherently limits its sensitivity due to the skin depth effect for surface or subsurface defect detection. To increase its penetration depth, it requires reducing its excitation frequency at the expense of coil sensitivity [5]. The multiple-frequency ECT [2] has been developed with simultaneous or sequential excitations for detecting defects at different depths and resolving the acquired signals that are affected by many variables, such as conductivity, permeability, geometry and probe's lift-off. The simultaneous excitation method results in a shorter testing time with less power consumption in each frequency component compared with the sequential excitation, which requires each system excitation to reach a steady-state before the next excitation. The swept-frequency approach [4] overcomes the multiple excitation problems with high precision and broad bandwidth potential for inspection of complicated areas by using a fixed probe. It has been applied for crack quantification [3], detection of thickness, measurement of permeability, and conductivity of materials coating [4,9,10] and object detection in more complex geometric areas [11]. However, the longer frequency sweep duration leads to lengthy inspection time scanning for defect positioning [12,13]. The pulsed ECT [5] has the potential for a shorter testing time with information of different depths due to its wide frequency bandwidth. It surpasses single- and multiple-frequency testing techniques due to its transient system response that potentially contains this wide spectrum of frequencies. It also contains, through the features, information on defect size, location and depth in the transient signal. However, it has lift-off variation including geometry and coupling between transmitter and receiver (Tx and Rx) coils, which carries no sample information. Moreover, lift-off effect by different normalization techniques has not effectively dealt with surface defects as effectively as with the subsurface, as the former presents similar signals to lift-offs [14].

The ECT works through a transmit–receive system by detecting induced eddy current-generated magnetic field from the material under inspection. The transmitter is usually made up of an induction coil, whereas the receiver could be using some magnetic field sensor. Traditional Tx–Rx coils have a high response and sensitivity to alternating flux linkage [15]. However, they are more sizeable than the defect and physically inflexible, which makes them possess lower spatial resolution and being prone to lift-off variations. Therefore, the quantification of natural defects in a metallic structure, especially with complex geometry like the curved surface in the pipeline, remains a challenging task.

Complex curved structures, like oil and gas pipelines, present a particular challenge during in-service inspection due to the variation of lift-offs. The problem is exacerbated for pipes buried underground. Even after detecting its defects with the proper equipment and excavating the defective section for accessibility, the pipe's surface can still be protected by coating and compressed sand. For inspecting such a structure without removing the coating, the probe has to be optimized for high lift-off inspection sensitivity at a certain coil gap [16]. However, this recent idea may only work effectively with flat and planar structures, where uniform lift-off can be easily achieved, which means it does not apply to curved surfaces. This issue can be resolved by using the flexible printed coil (FPC) array, where it has a fixed array of Tx and Rx coils on the same substrate. Its other benefit is that bending it to follow the curve of the surface does not affect their self and mutual inductances [17]. Hence, it is less influenced by lift-off variations and offers the benefits of wide area mapping capability with improved mutual inductance. Also, miniaturizing the FPC array provides micro-spatial resolution and lightweight capabilities for NDT&E applications for detecting and quantifying micro-defects in irregular metal structures [6,7,18]. For these reasons, flexible miniaturized coil arrays are used in our proposed ECT system.

The FPC array has been designed for curved surface structure inspection because of its potential advantages, such as good spatial resolution, high adaptability to different geometries and high efficiency with much higher sensitivity than the traditional flexible printed circuit [18]. Different FPC array configurations, such as single-coil array, pair of Tx–Rx array and single Tx with an array of Rx coils, were developed and investigated. The single Tx array has been used for measurements of tiny gaps between metallic and non-metallic surfaces [8]. Also, a pair of similar Tx and Rx flexible coils have been investigated for WPT efficiency on consumer electronics [17,19], and integrated smart textile and flexible fabric [20,21]. An array of Tx–Rx pairs of FPC, configured as a rosette eddy current array, were investigated for structural health monitoring and boosting the sensitivity of fatigue crack detection [22,23]. Instead of different pairs of Tx–Rx FPC coils, one excitation coil encircling an array of equally spaced Rx coils has been evaluated and found to be giving a higher response to defects due to improved mutual inductance and self-resistance [6,7,18]. The uniformly and equally structured fixed Tx array on differential and uniform Rx array for axial and tangential defects with a fraction of a millimetre depth has shown promising results based on a single-frequency ECT [24]. The axial and tangential defects are equally quantifiable by the FPC array. However, it suffers from mutual interference between neighbouring coils and sensitivity reduction at the expense of spatial resolution [18].

To overcome these challenges, a new method using the wireless power transfer (WPT) technology and the FPC array is proposed in this paper. The WPT technology has maximum energy transfer and a constant efficiency over a certain range [25,26], while the FPC array is adaptable to complex geometry for area mapping. This proposed WPT-based ECT differs from other ECT architecture due to its multiple resonance frequencies, each of which contains distinct information of possible defect signature. It uses swept-frequency excitation for obtaining different depth information and defect parameters. In this work, the advantages of the WPT-based ECT are combined with those of the FPC array for testing on curved structures. This work was previously implemented using traditional coils on artificial defects and a scanning method for multiple features, which was the first WPT for ECT [27]. The previously investigated features had two resonance characteristics, their two magnitudes and two features obtained from principal component analysis (PCA); hence, six features were used for the defect characterization. Now, the proposed work extends to the reconstruction of a dented area due to natural corrosion and metal loss in a pipeline sample using FPC arrays and feature fusion. The proposed probe contributes to ECT on complex structures, where scanning is not feasible for area mapping.

The rest of the paper is organized into five different sections. Section 2 describes the principles of the WPT concept as related to ECT and relevant theories of multiple feature extraction. Section 3 presents the experimental study through which the aim of the paper is achieved. Section 4 gives the results of multiple features analysis and discussion, while §5 gives the conclusion of the research achievements and future work.

## 2. Principles of the transmitter–receiver wireless power transfer system for the eddy current testing

A Tx–Rx WPT system comprises of an excitation circuit, two coils as a receiver (Rx) and a transmitter (Tx), and a load. The resonance property is achieved by series and/or parallel connection of multiple reactive elements like inductors and capacitors. The Rx and Tx can be configured as series–series (SS), parallel–parallel (PP) or a combination of series and parallel each with a compensating capacitor for efficient energy transfer [28,29]. As discussed in [28–30], different topologies have different impedance matching and quality factor equations that can lead to different measurement performance of WPT-based ECT systems. The PP resonant circuit was shown to have a higher sensitivity to metallic objects and a lower sensitivity to noise compared with the SS-resonant circuit due to its larger input impedance [31]. Therefore, the PP topology is selected for this study.

**Figure 1.**
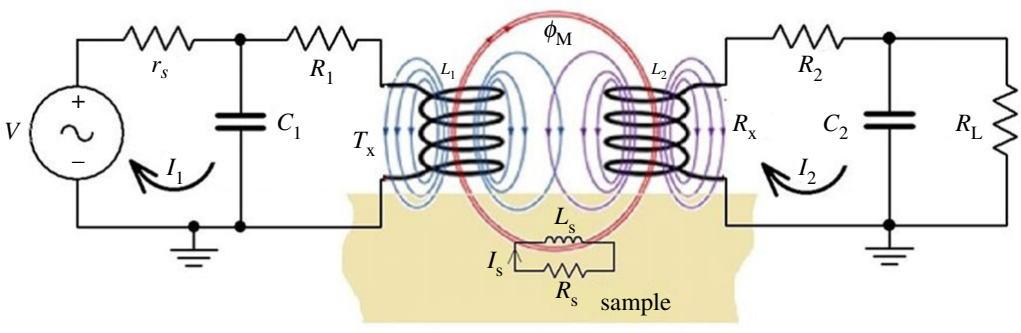
Equivalent circuit of Tx–Rx and metallic sample. (Online version in colour.)

Figure 1 shows the equivalent circuit diagram of the PP, Tx–Rx, WPT system including a metallic sample and an excitation voltage source *V* that is used in this paper. *I*_1_, *I*_2_ and *I*_S_ are the excitation currents through Tx's, Rx's induced current due to field linkage and sample's induced eddy current, respectively. Similarly, *R*_1_, *R*_2_ and *R*_S_ are the resistances of the Tx coil, Rx coil and the metallic sample, respectively. *L*_1_, *L*_2_ and *L*_S_ are the Tx, Rx and sample self-inductance, respectively. *C*_1_ and *C*_2_ are the compensated capacitors for Tx and Rx, respectively. The circuit in figure 1 can be evaluated using Kirchoff's laws to find the current flows through each element and voltage drop around each loop.

Kirchhoff's voltage law (KVL) states that the algebraic sum of all voltages around any closed loop in a circuit is zero. Applying KVL to the circuit shown in figure 1, the three loops in the circuit including Tx's excitation current, Rx's induced current and the sample's induced eddy current are represented by equation (2.1), where *M*_12_, *M*_1S_ and *M*_2S_ are the mutual inductances for Tx–Rx, Tx sample and Rx sample, respectively; each depends on their appropriate coupling coefficients and inductances.

2.1 $$\left(\begin{array}{c} V\\ 0\\ 0 \end{array}\right)=\left(\begin{array}{ccc} Z_{1} & -j\omega M_{12} & -j\omega M_{1\text{s}}\\ -j\omega M_{12} & Z_{2} & j\omega M_{2\text{s}}\\ -j\omega M_{1\text{s}} & j\omega M_{2\text{s}} & Z_{\text{s}} \end{array}\right)\left(\begin{array}{c} I_{1}\\ I_{2}\\ I_{\text{s}} \end{array}\right)\text{ , }$$

where the transmitter, receiver and sample units' equivalent impedances, *Z*_1,_ *Z*_2_ and *Z*_s_, respectively, are given as:

$$\begin{array}{rl} Z_{1} & =r_{\text{s}}+\left(\frac{1}{j\omega C_{1}}\right)//(R_{1}+j\omega L_{1}),\\ Z_{2} & =(R_{2}+j\omega L_{2})+\left(\frac{1}{j\omega C_{2}}\right)//R_{\text{L}},\\ Z_{\text{s}} & =R_{\text{s}}+j\omega L_{\text{s}}. \end{array}$$

From the last, the sample's loop KVL expression in equation (2.1), the expression for current, *I*_s_, is derived and given by (2.2). The derived value of *I*_s_ in (2.2) is substituted into Tx and Rx loop KVL in equation (2.1) to generate an equation model for Tx and Rx circuits in figure 1, which includes the effect of sample parameters. The circuits of Tx and Rx units are analytically described by the derived expression given by (2.3) and (2.5). The models given by (2.3) and (2.5) for Tx and Rx ports, respectively, described their self- and transfer impedances. The self-impedance of each port includes the effect of the nearby metallic sample as seen in figure 1:

2.2 $$\begin{array}{rl} I_{\text{s}} & =\frac{j\omega M_{1\text{s}}}{Z_{\text{s}}}I_{1}-\frac{j\omega M_{2\text{s}}}{Z_{\text{s}}}I_{2}\;\text{.} \end{array}$$

$$\begin{array}{rl} ∴V_{1} & =Z_{1}I_{1}-j\omega M_{12}I_{2}-j\omega M_{1\text{s}}\left[\frac{j\omega M_{1\text{s}}}{Z_{\text{s}}}I_{1}-\frac{j\omega M_{2\text{s}}}{Z_{\text{s}}}I_{2}\right] \end{array}$$

$$\begin{array}{rl} \Rightarrow V_{1} & =\left(Z_{1}+\frac{{\left(\omega M_{1\text{s}}\right)}^{2}}{R_{\text{s}}+j\omega L_{\text{s}}}\right)I_{1}-j\omega \left(M_{12}-M_{1\text{s}}\frac{j\omega M_{2\text{s}}}{R_{\text{s}}+j\omega L_{\text{s}}}\right)I_{2} \end{array}$$

2.3 $$\begin{array}{rl} V_{1} & =\left(Z_{1}+\frac{{\left(\omega M_{1\text{s}}\right)}^{2}(R_{\text{s}}-j\omega L_{\text{s}})}{{R_{\text{s}}}^{2}+{\left(\omega L_{\text{s}}\right)}^{2}}\right)I_{1}-j\omega \left(M_{12}-M_{1\text{s}}\frac{j\omega M_{2\text{s}}}{R_{\text{s}}+j\omega L_{\text{s}}}\right)I_{2}. \end{array}$$

The first part of *V*_1_ expression given by (2.3) is the voltage drop due to the new self-input impedance, *Z*_1_new_, of the Tx unit, while the second part is due to the reflected transfer impedance contributed by the Rx unit in the presence of the metallic sample. The affected parameter of *Z*_1_new_ is the actual Tx probe impedance, $R_{1}+j\omega L_{1}$, which is in parallel with the compensating capacitor, *C*_1_.

$$∴Z_{\text{Tx}\_\text{new}}=R_{1\_\text{new}}+j\omega L_{1\_\text{new}}=R_{1}+j\omega L_{1}+\frac{{\left(\omega M_{1\text{s}}\right)}^{2}(R_{\text{s}}-j\omega L_{\text{s}})}{{R_{\text{s}}}^{2}+{\left(\omega L_{\text{s}}\right)}^{2}}.$$

On rearranging $Z_{\text{Tx}\_\text{new}}$ and collecting the like terms, we have the new probe's impedance given by the following equation:

2.4 $$R_{1\_\text{new}}+j\omega L_{1\_\text{new}}=R_{1}+\frac{{\left(\omega M_{1\text{s}}\right)}^{2}R_{\text{s}}}{{R_{\text{s}}}^{2}+{\left(\omega L_{\text{s}}\right)}^{2}}+j\omega \left(L_{1}-\frac{{\left(\omega M_{1\text{s}}\right)}^{2}L_{\text{s}}}{{R_{\text{s}}}^{2}+{\left(\omega L_{\text{s}}\right)}^{2}}\right).$$

Similarly, by substituting the expression of *I*_s_ (equation (2.2)) in the Rx loop KVL equation (2.1), we have

2.5 $$0=-j\omega \left(M_{12}-M_{2\text{s}}\frac{j\omega M_{1\text{s}}}{R_{\text{s}}+j\omega L_{\text{s}}}\right)I_{1}+\left(Z_{2}+\frac{{\left(\omega M_{2\text{s}}\right)}^{2}(R_{\text{s}}-j\omega L_{\text{s}})}{{R_{\text{s}}}^{2}+{\left(\omega L_{\text{s}}\right)}^{2}}\right)I_{2}.$$

The first part of (2.5) is the reflected voltage drop from the Tx unit due to the transfer impedance, while the second term is the self-induced voltage across the Rx unit. By taking the second term, the new Rx self-impedance which includes the effect of the metallic sample, we finally derived the new resistance and inductance of the Rx coil as given by the following equation:

2.6 $$Z_{\text{Rx}\_\text{new}}=R_{2\_\text{new}}+j\omega L_{2\_\text{new}}=R_{2}+\frac{{\left(\omega M_{2\text{s}}\right)}^{2}R_{\text{s}}}{{R_{\text{s}}}^{2}+{\left(\omega L_{\text{s}}\right)}^{2}}+j\omega \left(L_{2}-\frac{{\left(\omega M_{2\text{s}}\right)}^{2}L_{\text{s}}}{{R_{\text{s}}}^{2}+{\left(\omega L_{\text{s}}\right)}^{2}}\right).$$

Equation (2.1) is used for determining the inductance and resistance in each of the resonant circuits as a function of the sample's and coil's parameters. The dominant coil's parameters are the self-inductance and -resistance which vary according to the sample conductivity, permeability and geometric nature due to eddy current interruption. The sample behaves as an inductor through which the eddy current circulates. The magnetic field generated by the induced eddy current in the sample affects the primary field linking Rx and, in turn, the equivalent parameters of Tx and Rx coils. The variations of effective resistance and inductance of the Tx and Rx coils as a result of the induced eddy current's influence on the Tx–Rx coupling depend on the excitation frequency, mutual coupling between the coil and the sample, and sample parameters. The relationship is derived from (2.1) and given in equations (2.7) and (2.8), which is similar to the model used for metallic object detection based on the WPT system [31–33]. The presence of the metal sample near the Tx or Rx increases losses due to the reduction in the magnetic field passing through the coil section as a result of the eddy current's effect in the sample. It then reduces Tx and Rx inductances as seen in (2.7), and hence, the resonance frequency point increases. Similarly, it increases the equivalent self-inductor resistance, which affects the voltage and current responses at the resonance point. However, at the point of defect, the voltage and current change slightly due to the variation in sample parameters as a result of the high influence of eddy current density around the defect area.

2.7 $$L_{\text{new}}=L_{i}-\frac{{\left(\omega M_{i\text{s}}\right)}^{2}L_{\text{s}}}{{R_{\text{s}}}^{2}+{\left(\omega L_{\text{s}}\right)}^{2}}$$

and

2.8 $$R_{\text{new}}=R_{i}+\frac{{\left(\omega M_{i\text{s}}\right)}^{2}R_{\text{s}}}{{R_{\text{s}}}^{2}+{\left(\omega L_{\text{s}}\right)}^{2}}\text{ , }$$

where *i* = 1 for Tx and 2 for Rx.

The new inductance and resistance values of Tx and Rx change the resonance point and the voltages across Rx and Tx. The change of input and output voltages at the resonance point contains the information about the sample's electrical conductivity, magnetic permeability and the defect parameters. The performance of a Tx–Rx WPT system is measured by its forward voltage gain, which is described by a transmission coefficient of the scattering parameters, *S*_21_, as defined by equation (2.9) [34–36]. It can be seen that the response depends on the sample's permeability, conductivity, geometry, the defect parameters and the operating frequency.

2.9 $$S_{21}=2\frac{V_{\text{L}}(\omega )}{V(\omega )}\sqrt{\frac{r_{\text{s}}}{R_{\text{L}}}}\;\text{.}$$

The Tx–Rx voltage ratio in equation (2.9) is derived by evaluating KVL on Tx and Rx circuits in figure 1 in the absence of a metallic sample. The derived voltage ratio of Rx output voltage across R_L_ and input voltage, *V*, as a function of frequency is given in equation (2.13). To derive the *S*_21_ response for the Tx–Rx WPT circuit in figure 1, the two-loop equations are generated from the Tx and Rx sides of the WPT circuit and Rx output voltage across the *R*_L_ resistor given by equations (2.10)–(2.12), respectively.

2.10 $$V(\omega )=(Z_{1})I_{1}-j\omega M_{12}I_{2},$$

2.11 $$0=-j\omega M_{12}I_{1}+(Z_{2})I_{2}.$$

2.12 $$\text{Using Ohm's}\;\text{law, }V_{\text{RL}}(\omega )=\left(\frac{1}{j\omega C_{2}}//R_{\text{L}}\right)I_{2}$$

$$∴\;\text{from}\;(2.11),I_{2}=\frac{j\omega M_{12}I_{1}}{Z_{2}}.$$

By substituting the expression of *I*_2_ in equations (2.10) and (2.12), we have

$$V(\omega )=\left(Z_{1}-j\omega M_{12}\left(\frac{j\omega M_{12}}{Z_{2}}\right)\right)I_{1}$$

$$\text{and}\;V_{\text{RL}}(\omega )=\left(\frac{1}{j\omega C_{2}}//R_{\text{L}}\right)\frac{j\omega M_{12}I_{1}}{Z_{2}}.$$

**Figure 2.**
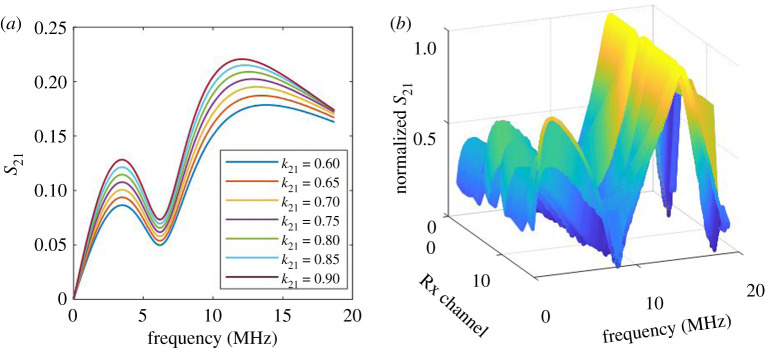
WPT system transmission coefficient, *S*_21_ responses. (*a*) Tx–Rx WPT system response for different coupling factors. (*b*) WPT-based ECT system sesponses for 16 Rx channels at a specific sample point. (Online version in colour.)

On substituting *Z*_1_ and *Z*_2_, in the expression of V(ω) and $V_{\text{RL}}(\omega )$, their ratio is given by the following equation:

2.13 $$∴\frac{V_{\text{RL}}(\omega )}{V(\omega )}=\frac{j\omega M_{12}\left(\left(\frac{1}{j\omega C_{2}}\right)//R_{\text{L}}\right)}{\left(r_{\text{s}}+\left(\frac{1}{j\omega C_{1}}\right)//(R_{1}+j\omega L_{1})\right)\left((R_{2}+j\omega L_{2})+\left(\frac{1}{j\omega C_{2}}\right)//R_{\text{L}}\right)+{\left(\omega M_{12}\right)}^{2}}\;,$$

where $V_{\text{RL}}(\omega )$ is the voltage across the Rx load (*R*_L_) as a function of the frequency, which depends on the eddy current's influence from the sample and the Rx's induced voltages. The mutual coupling between Tx and Rx, $M_{12}$, is mathematically defined by an expression $K_{21}\sqrt{L_{1}L_{2}};$ also, $r_{\text{s}}\;\text{and}\;R_{\text{L}}$ are the impedances of port 1 and port 2 of the vector network analyser (VNA) and their values are equal in our case. Therefore, the absolute values of the transmission coefficient, *S*_21_, over a certain range of frequencies can be plotted to give the frequency response behaviour of the Tx–Rx WPT system as seen in figure 2*a* for the model in the following equation:

2.14 $$∴S_{21}=2\frac{V_{\text{L}}(\omega )}{V(\omega )}\sqrt{\frac{r_{\text{s}}}{R_{\text{L}}}}=\frac{j4\pi fK_{21}\sqrt{L_{1}L_{2}}\left(\left(\frac{1}{j\omega C_{2}}\right)//R_{\text{L}}\right)}{Z_{1}Z_{2}+{\left(2\pi fK_{21}\sqrt{L_{1}L_{2}}\right)}^{2}}\sqrt{\frac{r_{\text{s}}}{R_{\text{L}}}.}$$

Similarly, the resonance frequency for the Tx and Rx circuits in figure 1 considered being equal for achieving a maximum power transfer efficiency and for the dual peak of *S*_21_ response to be symmetric about a resonance point, *f*_0_. It is theoretically obtained from the circuit in figure 1 by setting the imaginary part of the equivalent admittance of each Tx and Rx network to zero as a condition for a resonance point. The derived resonant frequencies are given by the following equations for Tx (*f*_0Tx_) and Rx (*f*_0Rx_), respectively.

2.15 $$f_{0\text{Tx}}=\frac{1}{2\pi }\sqrt{\frac{1}{L_{1}C_{1}}-\frac{{R_{1}}^{2}}{{L_{1}}^{2}}}\;$$

and

2.16 $$f_{0\text{Rx}}=\frac{1}{2\pi }\sqrt{\frac{1}{L_{2}C_{2}}-\frac{{R_{2}}^{2}}{{L_{2}}^{2}}}\;\text{.}$$

However, according to the circuit theories, analysis and experimental study, the Tx–Rx WPT response given by equation (2.14) has two split resonance frequencies in an over-coupled operation region [34,37]. The over-coupled region occurred at the higher mutual coupling between Tx and Rx coils, which depends on the distance between the two coils and their parameters. The interaction of the Tx–Rx coils with the sample affects the mutual coupling between Tx and Rx, proportional to the sample parameters. Equations (2.9) and (2.14) describe the response of the Tx–Rx probe system for the circuit given in figure 1. The coupling factor linearly depends on the Tx–Rx mutual inductance which depends on the sample influences as determined by equation (2.7). The designed values of the Tx–Rx parameters are given in table 1, experimental parameters and variables are used to compute the numerical model of *S*_21_ given by equation (2.14). Figure 2*a* shows *S* parameter of the reflected and transmitted signal values (*S*_21_) for different coupling coefficients (*K*_21_) of Tx–Rx as an indication of the response to the mutual coupling between the Tx–Rx and the sample. The two peak points of the response, *S*_21_, increases due to an increase of the mutual coupling between Tx–Rx coils. As discussed in [29], different topologies of magnetically coupled resonant Tx–Rx systems can be designed and developed through the selection of Tx–Rx capacitors *C*_1_ and *C*_2_. This extracts more power from the transmitter in the form of eddy current losses which has a significant impact on the system's parameters, especially for low-power applications [31,38]. For our FPC coil array, the Tx and each Rx mutual coupling results from the interaction with the metallic sample and defect.

**Figure 3.**
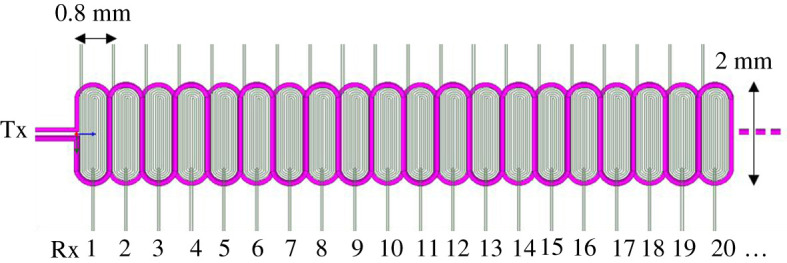
FPC array structure. (Online version in colour.)

**Table 1.** Experimental parameters and variables.

item	note	value
defect study	mapping a dent-area due to natural corrosion and metal loss in a class-A, American Water Works Association (AWWA) standard pit cast, cast-iron pipeline sample.	
transmitter, Tx	inductance, *L*_1_	1.26 µH
	equivalent resistance, *R*_1_	16.2 Ω
	resonant capacitor, *C*_1_	500 pf
	quality factor at *f*_0_	3.2
receiver, Rx array	each Rx has 0.8 mm spatial resolution, and all channels covered around 54 mm length	
	each Rx inductance, *L*_2_	1.9 µH
	each Rx equivalent resistance, *R*_2_	400 Ω
	resonant capacitor, *C*_2_ for each Rx	300 pf
	quality factor at *f*_0_	0.2
*f*_0_	design resonance frequency (air)	6.5 MHz
measuring instrument	E5071B, VNA with port 1 and port 2 having equal 50 Ω characteristic (*r*_s_ = *R*_L_ = 50 Ω)	
operating frequency	swept-frequency excitation (1601 frequency points)	300 kHz–19 MHz

Figure 2*b* shows the normalized values for measured *S*_21_ of the 16 Rx channels for the first measurement points on the pipeline surface. The two resonances in the ‘*M*’ shape response are not symmetrical because the FPC array configures one excitation coil with multiple received coils illustrated in figure 3, and the parameters are shown in table 1. Similarly, on considering a single Rx channel, the experimental transfer response resembles the analytical model presented in figure 2*a,b* for different Tx–Rx coupling factors, which are used as a measure of the mutual coupling with the sample.

The values in table 1 show the PP topology's parameters and their operating frequency range and quality factors for the study. Different resonance networks or components will be optimized in terms of feature selection and fusion to achieve optimal sensitivities and functionality of WPT-based ECT in future work.

In our proposed work, the mutual couplings of the FPC array are assumed to remain the same for bending it to sample surface geometry because Tx and Rx array are integrated on the same substrate. Therefore, the Tx and Rx array's mutual couplings are only affected by the influence of the metallic sample within their coverage area. Now, this paper will demonstrate the advantages of the WPT-based ECT systems using the FPC array, which include area mapping and scanning on a curved sample and multiple feature extraction, selection and fusion for defect characterization. The design, development and experimental implementations of the proposed system are described in §3.

## 3. Experimental studies

The system includes the probe, sample measuring instrument, data mining, feature imaging and defect characterization. The details of the sample used for the investigation are described in table 1. The probe is an FPC array fabricated on polyimide film with a thinner trace thickness and line spacing, which are advantageous for spatial resolution and sensitivity enhancement. The FPC array is made up of four layers with Tx covering the top and bottom layers, while 64 equally spaced similar Rx coils are distributed in the two middle layers. The coils, Tx and Rx arrays, composed of two parts each from different layers, are connected in series through vias [7]. The structure maximizes the mutual inductance between the Tx and Rx coil array, which then improves the Rx response and increases the signal-to-noise ratio. Figure 3 shows the Tx–Rx coil array used in this work, showing an excitation Tx coil surrounding a uniformly spaced and multiple detection Rx coil array. The Rx coils have identical inductance values which are 1.9 µH, while the Tx has an inductance of 1.26 µH, which were measured using HAMEG^®^ programmable LCR bridge HM8118 operating at 200 kHz. The Tx coil allows a considerable magnetic field to be evenly distributed across the Rx array. When the Tx coil is excited, it generates a spatially periodic magnetic field that induces a voltage in each Rx and eddy currents in the sample surface. According to the current continuity theorem, whenever there is a defect on the surface, the original flow path of the eddy current changes and flows around the edge of the defect. Hence, the generated eddy current field affects the primary field linking Rx, which manifests on the Rx's induced voltage carrying information on the sample and the defect.

A flexible sensor made of an array of receivers was used because of its advantages for area mapping at higher spatial resolution and flexibility to different geometric shapes [7]. First, 16 channels were used for dent-area inspection using a VNA to capture an ‘M’ shape response of Tx–Rx. The Tx and Rx channels were configured as parallel resonance network topology by connecting a capacitor in parallel as seen in figure 1. The values of *L*_1_, *L*_2_, *R*_1_ and *R*_2_ of the FPC array were inherent, while *C*_1_ and *C*_2_ were determined based on the resonance frequency, specific coil inductance and quality factors. The high resonance frequency is used for Rx channels for the inspection of surface dent defects. The parameters and values of the FPC array and their compensating capacitors are presented in table 1. The swept-frequency range is 300 KHz–19 MHz for the WPT ECT system. The optimal selection of the parameters of WPT topology and optimal operational frequency–dual resonance frequency will be discussed in our next paper.

**Figure 4.**
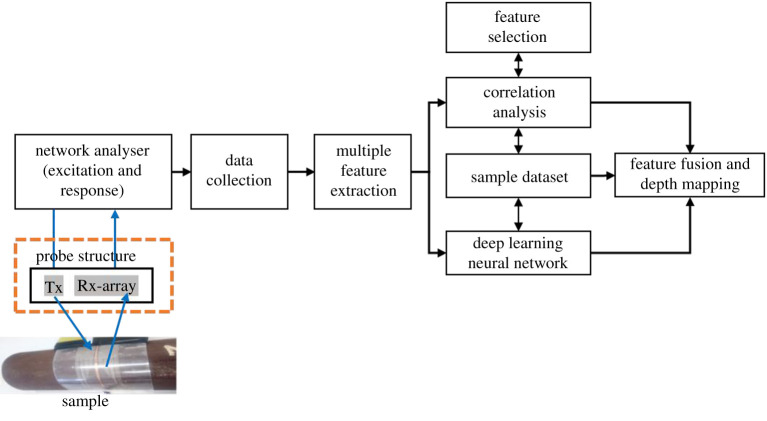
System block diagram of WPT-based flexible coil array ECT. (Online version in colour.)

**Figure 5.**
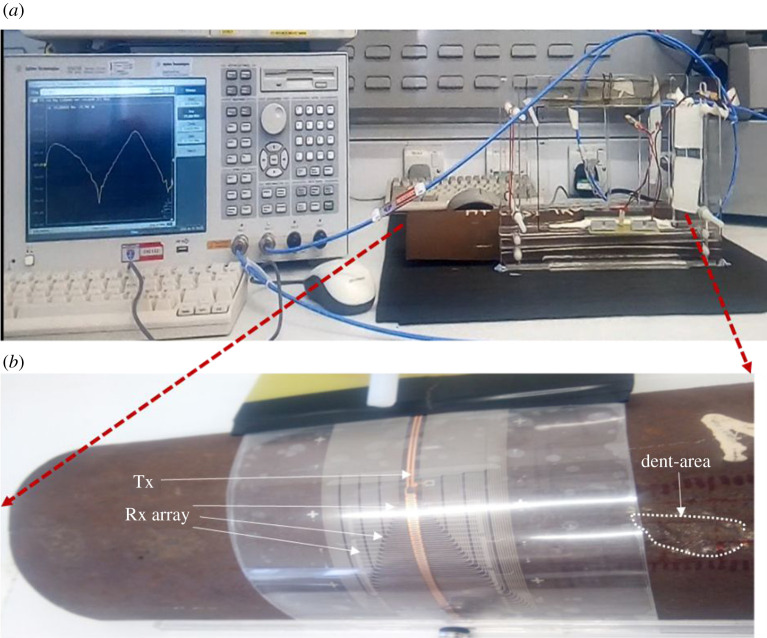
Experimental set-up and the FPC coil array over the pipeline sample. (*a*) Experimental set-up and (*b*) pipeline and probe. (Online version in colour.)

The system diagram is presented in figure 4. The system includes WPT-based ECT instrumentation using the VNA and FPC array, signal collection, multiple feature extraction, selection and fusion for quantitative non-destructive evaluation (NDE). The experimental set-up and the FPC coil array over the pipeline sample with natural dent defects are illustrated in figure 5, which is discussed in the next two paragraphs. The multiple feature extraction, feature selection and feature fusion are investigated in §4 with comparisons.

Figure 4 area is illustrated in figure 5*a* as an experimental set-up in the photo. Figure 5 shows the experimental set-up using the FPC array over a cast-iron pipeline (cut) sample with a natural dent from the industry. The Tx and each Rx coils were configured and connected to a VNA (VNA-E5071B) with Tx to port 1 and Rx to port 2. The Rx channels were connected sequentially, one at a time, because of the VNA connection constraint. The E5071B (300 kHz to 8.5 GHz) model has only two test ports. The VNA operates on a sweep signal, measures the transmission coefficients, *S*_21_, and displays the results on the screen. The Tx and Rx signals from the VNA ports are internally converted into an intermediate frequency signal by a mixer, then into a digital signal by an inbuilt analogue-to-digital converter (ADC) and finally sent to the processor. One ADC is available for each port signal, and the conversions take place simultaneously. Each of the inbuilt ADCs has a 16-bit resolution and a sampling rate of 570 k samples per second. Then, a microprocessor analyses the digital data and displays the results on the screen at a sweep speed of 9.6 µs/point. Finally, the computer unit is where the signal processing, features selection, extraction and processing for the defect evaluation take place.

The data measured by the VNA system, *S*_21_, have 1601 swept-frequency points among 300 kHz–19 MHz for every channel at a measurement point on the sample as illustrated in figure 5, the experimental set-up. For each measurement position of each C-scan, each of the 16 Rx channels covered 0.8 mm Y-spatial position and 2 mm X-spatial position with lift-off for the *Z*-spatial position. For our measurement of the dented area, the probe C-scan measurement was conducted by moving the sample axially at an equal interval of 2 mm for 40 mm distance for the defect to cross the probe. The first C-scan measurement point of 16 Rx channels, as presented in figure 2*b*, shows that the multiple responses with ‘M’ shape have multiple variables and features including lift-off for mapping to parameters. As illustrated in figure 2*b*, the ‘M’ shape response or double resonances have information related to the Tx–Rx probe-samples system relationship (lift-off and geometry), defect geometry and material inhomogeneity due to a wide range of frequencies. To investigate the capability of the proposed system for surface dent mapping and reconstruction, multiple features including resonant magnitudes and PCA were extracted on the dual resonance response and demonstrated in §4.

## 4. Multiple feature extraction, selection and fusion

The data for feature extraction, selection and fusion for mapping defect parameters are obtained and investigated using the proposed system response as shown in figure 2*b* and the dedicated cut pipeline sample with a natural dent. The dedicated dent sample is measured using a stylus profilometer. The depths of the dent are used for the comparison of multiple features in §4a, feature analysis and feature selection using cross-correlation analysis between the actual depths and extracted features in §4b, evaluation and comparison of feature fusion using deep learning and correlation method in §4c. Also §4c gives comparison of the highly correlated single feature from correlation analysis and the fused feature from the deep learning approach for best mapping to the actual depth of the dented area.

### (a) Multiple feature extraction

Extended from the previous work of multiple feature extraction of transient responses [39], four features are extracted and investigated from the ‘M’ responses, including the peak values of the two resonance peak points (*M*_1_ and *M*_2_), and PCA. The PCA is applied to each Rx dataset by subtracting each observation element from their corresponding observation average value and form a covariance matrix. Then, the eigen signals are computed from the eigenvalues, and the most significant signals are chosen according to the highest eigenvalues. The projections to spatial points of the chosen eigen signals are the extracted PCA features. The first PCA feature is applied for the two resonances. The two PCA features are PC_11_ and PC_21_ in the characterization of the ‘shape’ of the two resonance responses. The extracted features from each Rx channel at a point on the sample were first normalized to a range of [0,1] and then subtracted from its corresponding extracted features of a non-defect reference point presented in figure 2*b* to have normalized features.

**Figure 6.**
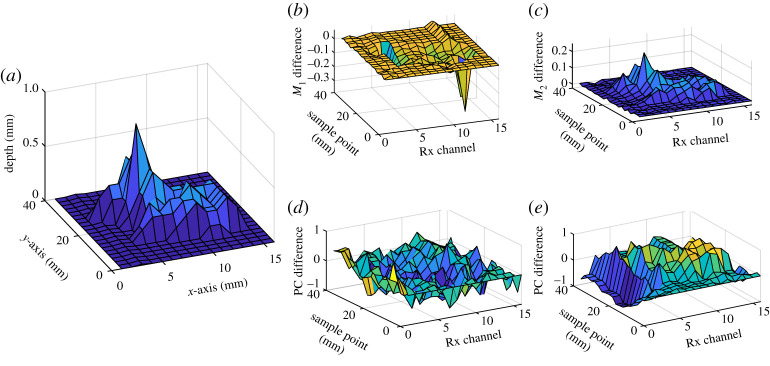
Actual dent profile and extracted features for (*a*) dent measured profile, (*b*) *M*_1_ feature of (first) peak value of Tx resonance, (*c*) *M*_2_ feature of (second) peak value of Rx resonance, (*d*) PC_11_ PCA feature of (first) Tx resonance principal component and (*e*) PC_21_ PCA feature of (first) Rx (second) resonance. (Online version in colour.)

The four features profile can be visualized and compared with the dent profile measured by a stylus profilometer in figure 6. The 3D plots in figure 6 use measurement positions of array spaces and C-scan as an Rx channel and the sample position in line with the *x*- and *y*-axis of the measured dent profile. Figure 6*a* illustrates the dent depth profile against the curvature surface; figure 6*b* illustrates the *M*_1_ feature of the peak values of Tx resonance (first resonance); figure 6*c* shows the *M*_2_ feature of the peak values of Rx resonance (second resonance); figure 6*d* shows *P*_11_ PCA feature of Tx resonance principal component (first); figure 6*e* illustrates *P*_21_ PCA feature of Rx (second) resonance. The *M*_1_ is negatively correlated to the actual depth of the dented area as it shows decreasing behaviour towards the dented area points. The *M*_2_ feature is positively correlated to the depth of the dented area as detected by Rx coils. As different features have different characteristics with the depth, a different feature extraction has a different reflection of defect characters. None of the extracted features reflect the defect contour with a good correlation. A comparison of different feature correction with defect depths for feature analysis, feature selection and feature fusion using correlation and deep learning methods for defect mapping are investigated in the next section.

### (b) Feature analysis and selection for defect depth

**Figure 7.**
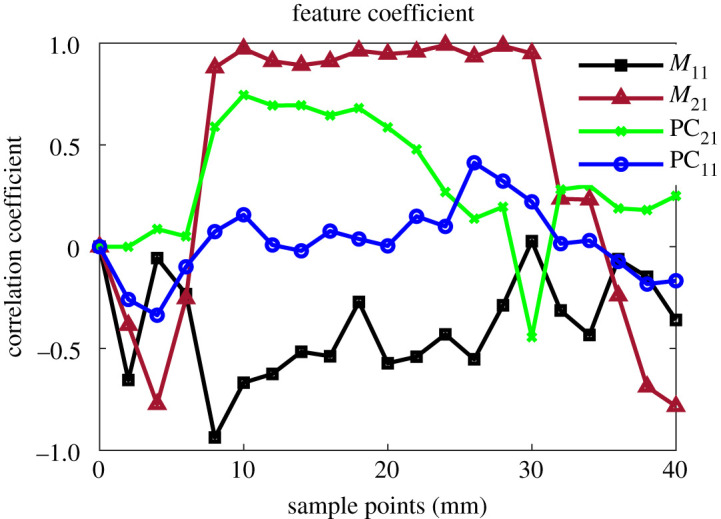
Feature correlation with the sample dent depths for all measurement points. (Online version in colour.)

The extracted multiple features have different characters of the defect depths as illustrated in figure 6. The relevant features for defect depth information need to be selected and fused for 3D defect mapping and reconstruction. The correlation method is used for multiple feature analysis and selection. The correlation coefficients for a set of features at every measurement point give a measure of its relevancy to the actual depth parameter. The cross-correlation between actual depths of the dented area along with the Rx array (*x*-axis) and the extracted features at every measurement point (*y*-axis) was evaluated. The set of correlation coefficients between each array of the extracted feature and the depths of the dented area for each measurement point were obtained. Figure 7 shows the extracted features' correlation, each with its dedicated depth at each measurement point. A feature coefficient of ±1 indicates a perfect degree of correlation with the defect parameter. The positive correlation by *M*_2_ and PC_21_ and negative one by *M*_1_ features were due to the behaviour of the WPT system's dual response towards the two peak resonance values and overall response shape as shown in figure 2*b*. The first resonance peak *M*_1_ decreases, while the second peak *M*_2_ increases as the distance to the dent decrease, which are caused by decreasing mutual coupling between Tx–Rx coils. PC_11_ has the lowest correlation, whereas the second magnitude *M*_2_ has the highest correlation coefficient with the defect depth due to the Rx response with the sample and defect. It can be applied for a single feature application. It is understandable, one single Tx excitation and multiple Rx illustrate location information during NDT&E without scanning. For using multiple features, correlation-based and deep learning-based feature fusions are applied and compared in this study.

### (c) Feature fusion for defect depth and 3D mapping

In this section, two feature fusion approaches including deep learning-based fusion and canonical correlation analysis (CCA)-based fusion are used to strengthen the WPT-based ECT's capability for mapping the defect area. The quantitative analyses of different feature extractions and fusion strategies are conducted in terms of *R*^2^ value and mean square error (MSE) values between defect parameters and features. The value of *R*^2^ is a statistical measure of data fit that indicates the proportion of the variation of dependent variables described by the independent variables in a regression model, whereas the MSE represents how close a regression line is to a set of points.

**Figure 8.**
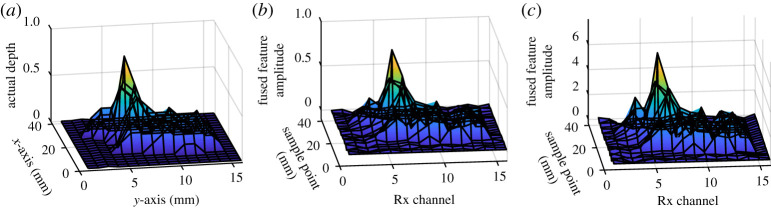
Fused features mapping to depths at different measurement points: (*a*) dent measurement profile, (*b*) fused feature by deep learning and (*c*) fused feature by the CCA. (Online version in colour.)

To implement the deep learning-based feature fusion for the four extracted features for defect 3D mapping, this work applied the two-layer feed-forward deep learning network to build the learning model between features and defect depths. The first 50% of the features and actual depths data served as training, and the rest of 50% is used for validation using the trained neural network model. Four features each with the same dimension *F*_16×21_ were reshaped to 1D signal *F*_1×336_. Thus, the whole feature set is *F*_4×336_ and serves as an input for the network. The target to train the model is real defect depth parameters with the same dimension of 1 × 336. The model used a Levenberg–Marquardt technique for optimizing the network structure at different iterations. The MSE of the training set is lower than 0.001 and that of testing and validation set is lower than 0.01, which indicates that the model can well describe the relationship between four features and the defect depths. After validation of the deep learning model, the whole four feature sets including *M*_1_, *M*_2_, PC_11_ and PC_21_ are fused by the model, mapping the 3D profile of the defect shown in figure 8*b*.

To validate the deep learning techniques' capability for feature fusion, the feature fusion was also achieved using the CCA [40–43]. The CCA combines multiple datasets into a common representation across subjects for denoising and dimensionality reduction. The CCA forms a linear combination from both datasets for maximizing an individual variable's weight and effectiveness of the parameter set as a whole. In this work, the CCA-weighted factors were calculated between defect depths and individual feature sets. Then, each feature set is multiplied by the weighted factors and summed together to obtain the CCA fusion feature.

It is observed that the true defect profile as shown in figure 7*a* has a good agreement with fused features in figures 8*b* and 8c. There are only minor differences seen in non-defect areas. To quantitatively evaluate the fused features' capability for mapping the defect areas, the *R*^2^ value, which is the square of the correlation between the defect parameters and the fused features, and the MSE are used for different feature sets. It is shown that in table 2 and figure 8, the fused features by the deep learning network have the highest *R*^2^ value and the lowest MSE value, proving it is the best feature for mapping the defect using the WPT-based ECT system. The CCA fusion feature shows reasonable *R*^2^ and MSE values. Thus, it is well understood that fusion features including deep learning and CCA can merge the behaviour of sub-features and show their capability for defect mapping and reconstruction. Besides the fused features, *M*_2_ (second resonant magnitude) also has an *R*^2^ value of 0.9104 and an MSE value of 0.0016, indicating the frequency band 14.5–16.0 MHz of the system is sensitive to surface dent. PC_11_ and PC_21_, from the two resonances, can also illustrate that Rx resonance has better depth responses than Tx resonance as resonance peak values. The fusion of multiple features with different weightings by using deep learning and correlation methods has better performance of depth estimation than a single feature in terms of *R*^2^ and MSE values. The deep learning method for the multiple feature fusion has the best performance of depth prediction.

**Table 2.** *R*^2^ and MSE values for the comparison of extracted and fused features.

features for the characterization of defect depths	*R*^2^	MSE
deep learning-based feature fusion	0.9253	0.0013
CCA-based feature fusion	0.9171	0.0015
*M*_1_	0.2755	0.0143
*M*_2_	0.9104	0.0016
PC_11_	0.2690	0.0135
PC_21_	0.4318	0.0147

## 5. Conclusion and future work

In this paper, WPT and flexible coil array were integrated for the ECT of a pipeline sample with a dented area due to metal loss and corrosion. The dual resonance response *S*_21_ of the integrated system was investigated for multiple feature extraction, selection, fusion and mapping for a 3D defect reconstruction and characterization. The experimental system of WPT-based eddy current NDT using the FPC array has demonstrated its feasibility for NDT&E of curved surface. The multiple responses with double resonances can provide multiple features for depth characterization. The extracted feature correlation analysis shows that the feature of the second resonance peak values has the highest correlation with the depth profile of the dented area for having the highest *R*^2^ value and the lowest MSE value. It can be explained that the FPC array has one excitation coil (Tx) and multiple received coils (Rxs), and the second resonances from Rxs have better local information. This demonstrates the capability of the Rx resonance unit on responding to eddy current losses in the sample and defect compared with the Tx resonance point. Also, the deep learning has shown to surpass the canonical correlation analysis in the feature fusion for the 3D reconstruction of the dent, as the former has the highest *R*^2^ value and the lowest MSE value.

Based on the proposed multiple feature extraction and fusion for defect mapping, further optimization of comparison of topologies of the WPT-based ECT system including a selection of WPT topologies, coils and capacitance parameters, their equivalent quality factors [29,30] and their performance for sensitivity and functionality of quantitative NDE needs to be further investigated. The results are only given to the depths of surface dent-area characterization of complex metallic structures inspection. The future work can extend the proposed work of WPT-based ECT systems and multiple feature extraction, selection and fusion for different types of defects and their parametric estimation like rolling contact fatigue, stress corrosion crack and other hidden defects [44,45]. Also, the proposed system will be miniaturized using WPT IC chips for operating at optimal frequency ranges. It can be implemented as a portable system or a permanently installed sensor system for *in situ* structural health monitoring.

## Data accessibility

This article has no additional data.
